# Association between Stress and the HPA Axis in the Atopic Dermatitis

**DOI:** 10.3390/ijms18102131

**Published:** 2017-10-12

**Authors:** Tzu-Kai Lin, Lily Zhong, Juan Luis Santiago

**Affiliations:** 1 Department of Dermatology, Kaohsiung Chang Gung Memorial Hospital and Chang Gung University College of Medicine, Kaohsiung 83301, Taiwan; 2 Citrus Valley Medical Center, West Covina, CA 91790, USA; lzhong5@calstatela.edu; 3 Dermatology Service & Translational Research Unit (UIT), Hospital General Universitario de Ciudad Real, Ciudad Real 13005, Spain

**Keywords:** atopic dermatitis, HPA axis, psychological stress, skin barrier, inflammation, glucocorticoid

## Abstract

The hypothalamic–pituitary–adrenal (HPA) axis is one of the body’s neuroendocrine networks that responds to psychological stress (PS). In the skin, there exists a peripheral HPA axis similar to the central axis. Glucocorticoids (GCs) are key effector molecules of the HPA axis and are essential for cutaneous homeostasis. Atopic dermatitis (AD) is a condition typically characterized by a chronic relapsing course that often results in PS. HPA dysfunction is present in AD patients by the decreased response of GCs elevation to stress as compared to those unaffected by AD. Nevertheless, in skin, acute PS activates several metabolic responses that are of immediate benefit to the host. During the acute phase of PS, increased endogenous GCs have been shown to provide benefit rather than by aggravating cutaneous inflammatory dermatoses. However, a chronic T helper cell type 2 (Th2) predominant cytokine profile acts as a negative feedback loop to blunt the HPA axis response in AD. In this article, we reviewed the role of CRF, pro-opiomelanocortin (POMC)-derived peptides, GCs of the HPA, and 11β-hydroxysteroid dehydrogenase 1 (11β-HSD1) in AD, with a discussion of the pathogenetic mechanisms of inflammation and skin barrier functions, including antimicrobial defense, and their association with PS.

## 1. Introduction

Atopic dermatitis (AD), one of the most common chronic allergic skin inflammatory diseases, has an increasing prevalence in the population. Onset is typically during the first six months of life in 45% of cases, and the symptoms may continue to affect adulthood [1]. Current evidence suggests that the pathogenesis of AD is multifactorial, comprising not only aspects of epidermal barrier dysfunction, but also T helper cell type 2 (Th2)-lymphocyte-driven inflammation [2,3]. The disease is also modified by neuroendocrine mediators such as histamine via histamine H4 receptor [4] or glucocorticoids (GCs) of the hypothalamic–pituitary–adrenal (HPA) axis [5]. It is well known that AD is triggered or exacerbated by psychological stress (PS) [1,3]. In both pediatric and adult patients, AD often causes significant impairment in quality of life, resulting in PS. PS, via the HPA axis, leads to the release of endogenous GCs that is critical in regulating the inflammation of AD [5].

**Figure 1:**
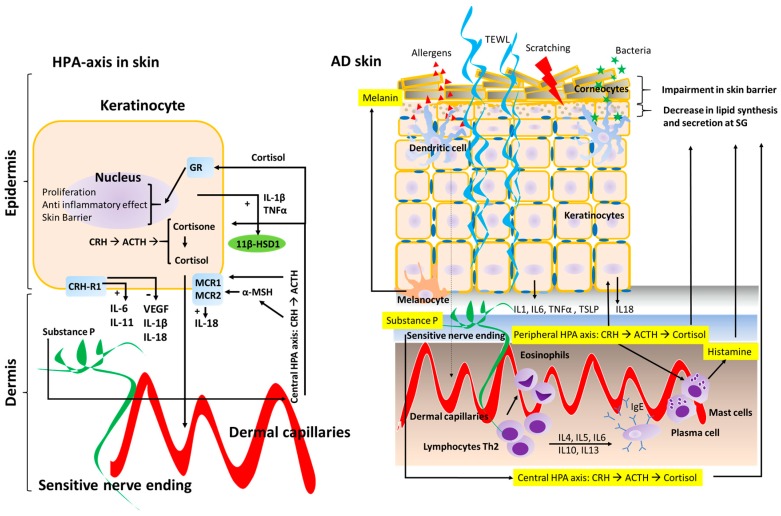
Schematic representation of the Hypothalamic-Pituitary-Adrenal (HPA) axis in the skin. The effects on keratinocytes (left diagram) on AD skin in regards to skin barrier structure and inflammatory response (right diagram). The central HPA axis and the peripheral HPA axis both regulate skin barrier homeostasis and the inflammatory response in the skin. Peripheral nerve endings in the skin are represented in green, whereas dermal capillaries are shown in red. Transepidermal water loss (TEWL) is represented by curled blue lines (right diagram). Corticotrophin-releasing hormone receptor type 1 (CRH-R1); Glucocorticoid receptor (GR); Melanocortin receptor type 1 and 2 (MCR1 and MCR2); 11 beta-hydroxysteroid dehydrogenase 1 (11β-HSD1).

Impairments in skin barrier function are inevitably present in AD patients. In infants, transepidermal water loss (TEWL) has been shown to be a strong predictor for developing AD later in life given its reflection of stratum corneum (SC) structure and permeability barrier function. In fact, impairment of the skin barrier is considered a primary event in AD pathogenesis [3] (Figure 1). It has been shown that TEWL is correlated to both allergic and irritant contact dermatitis [6], and perturbed barrier function plays an essential role in the allergic sensitization to both protein antigens and staphylococcal superantigens in AD patients [7]. Additionally, perturbation of epidermal barrier function correlates with initiation of cytokine cascade in human skin [8]. Therefore, it has been proposed that early interventions to repair this epidermal barrier including the use of appropriate emollients, humectants, and soaps, may be useful in the control of this chronic disease and the prevention of its progression (atopic march) [9]. Several studies have evaluated the impact of inflammatory mediators (cytokines, hormones, and antimicrobial peptides (AMPs)) on AD skin lesions, and their influence on barrier function and B-cell–mediated immunoglobulin production [10]. Th2-related cytokines such as interleukin 4 (IL-4) and IL-13 play a significant role in AD inflammatory response. Tumor necrosis factor alpha (TNF-α), the most studied cytokine in skin physiology, is increased in both skin lesions and the serum of AD patients. TNF-α is also associated with increased histamine concentration in plasma, which has been thought to also be involved in the pathophysiology of AD [11]. Moreover, skin irritants or environment noxious stressors (low humidity, heat, PS, etc.) lead to the release of IL-1 and TNF-α, which affect the function of antigen-presenting cells and promote the development of contact allergy. These findings may explain the positive association between contact allergy and AD, which has been established in prior epidemiological studies [12].

Thymic stromal lymphopoietin (TSLP), a sensitive marker of epithelial inflammation, is shown to be harmful to the epidermal barrier [13,14,15,16]. In fact, TSLP is highly expressed in keratinocytes from AD patients, and plays critical roles in the induction and exacerbation of this chronic dermatitis [17]. TSLP acts in AD pathogenesis through several mechanisms: (1) enhancing CD4+ T-cell polarization into Th2 cells [16,18,19,20,21]; (2) promoting IL-33-independent innate lymphoid cell responses [22]; and (3) targeting dendritic cells and innate lymphoid cells [23,24]. Moreover, TSLP mediates pruritus, which is not only the main symptom of AD, but also a key factor involved in its pathogenesis by causing disrupted skin barrier through scratching and exposure to pathogens, thus leading to the consequent inflammatory flares [25,26].

Finally, neuroendocrine mediators also play an important role in AD pathogenesis, with the HPA axis being one of the most well studied and best characterized [27,28,29]. HPA dysfunction is present in AD patients, showing a decreased response of serum GCs after being exposed to stressors when compared with unaffected persons [27,28]. Although the HPA axis is initially activated by pro-inflammatory cytokines, there is attenuated GC responsiveness to stress in chronic AD [29]. Thus, in patients with AD, insufficient production of the GCs might result in an imbalance of Th1/Th2 and trigger allergic inflammation [30,31]. This review has focused on understanding the complex interplay between the HPA axis, inflammation, skin barrier function, and PS in AD.

## 2. Hypothalamic–Pituitary–Adrenal (HPA) Axis

**Figure 2:**
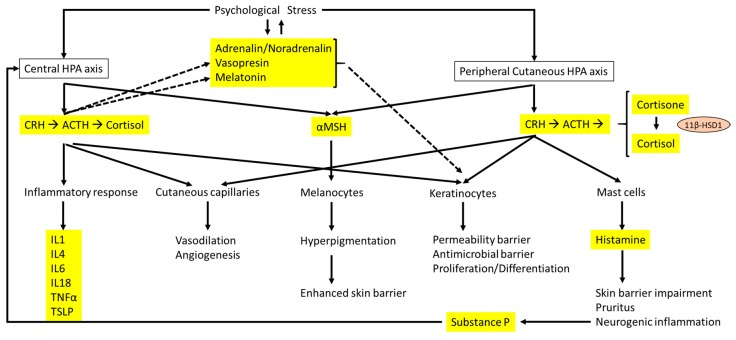
Interactions between the central HPA axis and the cutaneous HPA axis and the effects on the skin barrier and dermatitis. The main hormones, neurotransmitters, and cytokines are highlighted in yellow. Solid arrows represent direct effect of hormones and neurotransmitters related to the HPA axis, whereas dashed lines indicate the influence of psychological stress on other hormones and neurotransmitters that are different from the HPA axis and linked to epidermal homeostasis.

The HPA axis (Figure 2) has very important functions, including responses to psychological and physical stress, and inflammatory factors [32]. Corticotrophin-releasing hormone (CRH) is the principal component of the HPA axis, functioning as the key regulator of stress response. CRH is released from paraventricular nuclei of the hypothalamus in pulsatile pattern, with the increase in magnitude and frequency secondary to acute stress [33]. This hormone controls the HPA axis, as well as various behavioral and autonomic stress responses by the two CRH receptors, CRH-R1 and CRH-R2. The CRH system also includes CRH-binding protein, a glycoprotein that binds to and modulates CRH receptor activity [34]. Additionally, CRH regulates both systemic and cutaneous homeostasis [35], inducing the secretion of pro-opiomelanocortin (POMC)-derived peptides from the anterior pituitary, including adrenocorticotropic hormone (ACTH), alpha melanocyte-stimulating hormone (α-MSH), and β-endorphin [33]. ACTH shows a direct effect in pigmentation [36]. α-MSH has strong anti-inflammatory effects [37], and β-endorphin exhibits analgesic, immunomodulatory, and anti-inflammatory properties [38]. During PS, activation of HPA axis has been correlated to increased levels of pro-inflammatory cytokines (IL-1, IL-4, IL-6, IL-18, and TNF-α), probably through the action of ACTH [39]. In addition, it is well known that ACTH mediates the inflammatory response by stimulating adrenal GC production (cortisol in human), which act in many cells and tissues to regulate homeostasis [33]. As important effector molecules of the HPA axis, GCs are regularly secreted, with feedback inhibition modulating the HPA axis [40]. GCs influence the transcription of glucocorticoid response elements of target genes involved in anti-inflammatory response [41]. GCs are key mediators to modulate the inflammatory response in PS. In fact, one of the main pathogenetic mechanisms of chronic allergic diseases, such as AD or asthma, is the presence of greater Th2 response correlated to lower cortisol and GC levels [42,43,44].

## 3. The Peripheral HPA Axis

The skin has developed a local neuroendocrine system to regulate its homeostasis in response to its ongoing exposure to environmental stressors [45,46]. In fact, components of this neuroendocrine system have been described for many structural cells of the skin, although they have been particularly well characterized for keratinocytes [47,48,49,50]. Specifically, there exists a peripheral HPA axis similar to the central HPA axis, which has been recently mapped [38,51,52]. This peripheral HPA axis also shows a similar hierarchical structure to the central axis. It has been reported that all regulatory elements of the central HPA axis were expressed in mammalian skin, including CRH, POMC-derived peptides, GCs [36,53], and related peptides, as well their appropriate functional receptors, CRH receptors, melanocortin receptor type 2 (MCR2, the classical adrenocortical ACTH receptor), and GC receptor NR3C1 [54,55].

The peripheral HPA axis acts in an auto/paracrine manner, modifying the cutaneous stress response. Not only are keratinocytes able to produce hormones like CRH, ACTH, and cortisol, but they also produce neurotransmitters (e.g., adrenaline, noradrenaline, dopamine, histamine, acetylcholine, etc.), neurotrophins (e.g., nerve growth factor, brain-derived neurotrophic factor), as well as neuropeptides that also respond to stress, e.g., substance P [38,39,45,46,47,48,49,50]. In addition to synthesizing neurotransmitters and neurohormones, keratinocytes also express their respective receptors [38,39,45,46,47,48,49,50,56,57]. Thus, the epidermis continuously senses the environment and reacts to various stressors (humidity, temperature, skin surface pH, cutaneous microbiome, injuries, and PS) to maintain epidermal homeostasis and adjust skin barrier functions [36,38,39]. Of the various stressors, PS plays a key role in skin barrier and influences the immune response by the secretion of stress-related neuropeptides/cytokine profiles and altering the HPA axis-related hormones [39,53,58].

Unlike the central HPA axis, the elements of peripheral HPA axis (CRH→POMC→ACTH→GCs) affect each other more closely and have more complex role. This is still not well understood [35]. Of all the elements of the HPA axis, CRH function is well studied [38,39,59]. However, it is still unclear if CRH in the skin is produced centrally or locally [59]. The effect of CRH is variable on different types of cells. Peripheral CRH is an important proinflammatory cytokine necessary to induce an inflammatory response in vivo, leading to increased skin vascular permeability and inflammation, which is largely attributed to mast cell activation [60]. CRH function is mediated via CRH receptors (CRH-R1 and CRH-R2) [52]. CRH-R2 is mainly present in vascular and glandular structures while CRH-R1 is the major receptor of most cellular constituents in the skin [52,61]. Through CRH-R1, CRH influences the proliferation [62], differentiation [63,64], and apoptosis [62] of the various cell components in the skin. CRH promotes the differentiation of keratinocytes and inhibits the proliferation through arrest in G0/1 phase [63,64]. CRH also extends the melanocyte survival by suppressing apoptosis [62], but inhibits their proliferation. Meanwhile, CRH stimulates the proliferation of fibroblasts [62]. In regard to the effects on inflammation, CRH promotes the release of IL-6 from keratinocytes [65]. It also stimulates mast cells and plays a role as a pro-inflammatory agent [60,66]. In contrast to its role of proinflammatory effects, CRH also shows its anti-inflammatory characteristics by modulating vascular permeability, angiogenesis, and cytokine production [65,67,68]. Through CRH-R1 activation, keratinocytes reduce the expression of vascular endothelial growth factor [68]. CRH also decreases the pro-inflammatory cytokine IL-1β expression of keratinocytes, but increases the expression of the anti-inflammatory cytokine IL-11 [65]. In addition, in human melanocytes, CRH inhibits nuclear factor-κB signaling and the subsequent production of pro-inflammatory cytokines [69].

GCs are the last mediators in peripheral HPA axis. They are also synthesized in the skin [38,39,45,46]. 11 beta-hydroxysteroid dehydrogenase (11β-HSD) plays a role in GC synthesis. Two isozymes of 11β-HSD exist: 11β-HSD1, which activates cortisol from cortisone, and 11β-HSD2, which inactivates cortisol to cortisone [70,71,72]. 11β-HSD1 is expressed in many tissues, with the highest quantities in the liver, lung, adipose tissue, ovaries, and the central nervous system [71]. In the skin, 11β-HSD1 is expressed in epidermal keratinocytes, dermal fibroblasts, and root sheath cells of the outer hair follicle [73,74,75]. Specifically, in a wound healing context, 11β-HSD1 activates significantly more corticosterone than is generated de novo from progesterone in mouse skin, driving GC exposure [76]. This feature has been shown to accelerate skin wound repair with the use of 11β-HSD1 inhibitors [76,77]. Topical applications of carbenoxolone (a nonspecific 11β-HSD inhibitor) or an isoform-specific 11β-HSD1 inhibitor have been shown to overcome stress and exogenous GC-induced delays in the wound healing process [77].

## 4. HPA Axis and Skin Inflammation

As previously discussed, the HPA axis can be stimulated by a multitude of environmental stressors, including inflammation, infections, and direct physical trauma (scratching, wound healing, burns, etc.) [78]. Moreover, several studies have found proof suggesting communication between proinflammatory cytokines and mediators of the HPA axis [79,80,81,82]. Most reports investigating the link between elevated PS and worsened inflammation in humans have shown that both the HPA axis and the sympathetic axis contribute to the distinctively large volume of pro-allergic cytokines (e.g., IL-4 and IL-5), which drive eosinophilic inflammatory response and determine the inflammatory immunophenotype in AD patients. Additionally, there is also an up-regulation of cytokines driving cellular adaptive immunity, including TNF-α and interferon gamma (IFN-γ) [39,83,84]. IL-18 is a proinflammatory cytokine with an important role in PS by modulating the HPA axis’s effects on the adrenal and pituitary glands in the presence of stressors [39]. In skin, keratinocyte production of IL-18 is known to trigger severe cutaneous inflammation. ACTH promotes IL-18 secretion through caspase-1 activation [81], whereas CRH down-regulates IL-18 expression through activation of the p38 MAPK pathway [82]. Thus, IL-18 contributes to the CRH negative feedback loop.

Research has been also conducted on POMC to evaluate its role in skin inflammation. POMC and POMC-derived peptides function by binding MCR1 and MCR2, with MCR1 being the major receptor in keratinocytes, melanocytes, and adipocytes [85]. α-MSH, for example, exhibits anti-inflammatory properties and stimulates skin pigmentation through MCR1 [85].

GCs are major components in the inflammatory response through GC receptor (GR) binding. Research on mice with keratinocyte-restricted GR inactivation (GR epidermal knockout or GR (EKO) mice) has shown that epidermal loss of GR leads to skin barrier defects and susceptibility to cutaneous inflammation. Moreover, macrophage influx and mast cell degranulation have been observed in newborn GR (EKO) skin, both of which are hallmarks of AD [86]. GC synthesis is also a key process in the peripheral HPA axis modulation. There has been evidence that 11β-HSD1, the main enzyme involved in cortisol synthesis, is induced in vitro by the pro-inflammatory cytokines IL-1β and TNF-α. Increased cortisol concentration has also been noted in culture media [70]. Thus, it has been hypothesized that the regulation of cytosolic cortisol concentrations by 11β-HSD1 may modulate inflammatory cytokine expression in keratinocytes [70]. 

## 5. HPA Axis and Skin Barrier

In response to various stressors, sensory nerves or humoral factors activate the central and peripheral cutaneous HPA axis to regulate homeostatic responses. These responses serve to counteract cutaneous and systemic environmental damage in order to maintain cutaneous homeostasis [35].

ACTH is involved in melanin production, which protects skin from ultraviolet irradiation. Melanin has been recently found to correlate to the barrier function. Keratinocytes with more melanin display superior barrier function in comparison to lightly-pigmented keratinocytes in organotypic human keratinocytes [87]. In a study of homogenous Chinese population, the epidermal barrier function was inferior in stable, non-inflamed, depigmented (vitiliginous) skin in comparison to adjacent, pigmented skin sites in the same individuals [88]. Hence, through the increased melanin production, ACTH supports preservation of epidermal barrier function (Table 1). On the other hand, β-endorphin also protects skin function by enhancing epidermal turnover rate [89].

Topical and systemic GCs are two of the most effective classes of drugs for treating acute and chronic cutaneous inflammatory dermatoses. The effectiveness of topical GCs in the treatment of AD has been well established [90]. In the skin, GCs target keratinocytes and inflammatory cells in the dermis, where they exhibit their major anti-inflammatory mechanism by suppressing of genes related to transcription factors, e.g., nuclear factor-κB and activator protein-1 [91]. However, additional mechanisms may be involved in the anti-inflammatory response. Studies have shown that topical GCs improve AD-like skin lesions and barrier impairment by suppressing TSLP-related allergic inflammation [92,93]. GCs inhibit TSLP release from keratinocytes in an atopic cytokine environment [93]. However, GCs also affect proliferation, differentiation, and metabolism of keratinocytes [94]. This could possibly compromise the skin barrier function in the long run, resulting in exacerbation of AD. Moreover, prolonged and excessive use of potent topical GCs may contribute to short-term hormonal changes in the central HPA axis [95].

The main endogenous GC found in humans is cortisol, which is produced by the adrenal cortex of the central HPA axis and keratinocytes of the peripheral cutaneous HPA axis. The enzyme 11β-HSD1 acts to activate the epidermal cortisol production in keratinocytes [96]. Cortisol production is increased during wound healing in the epidermis. Environmental dryness might also induce increased cortisol secretion in epidermis of diseased skin [97], which could potentially influence a patient’s psychological well-being and systemic physiology. A study of pulp development in a murine knockout model has demonstrated that fetal GC deficiency delays SC maturation through the reduced expression of the proteins involucrin, loricrin, and filaggrin, and the reduced activity of the lipid synthetic enzymes beta-glucocerebrosidase and steroid sulfatase [98].

## 6. The Effect of Stress in AD on the HPA Axis

Many chronic skin disorders, including psoriasis, seborrheic dermatitis, rosacea, and AD are adversely affected by PS. However, the responsible mechanisms leading to these pathologies are not yet clearly understood [99]. Through the HPA axis, the immunological changes caused by an endogenous neuroendocrine stress response are different from the immunosuppressive changes resulting from therapeutic GC dosages applied in dermatology. A decreased response from the HPA axis in AD patients is thought to result from the disease itself rather than from topical GC application [100,101]. Some reports have suggested that a chronic Th2-predominant cytokine profile acts as a negative feedback mechanism to decrease the HPA axis response. Accordingly, it was observed that IL-4, a Th2 cytokine, directly inhibited POMC expression in the anterior pituitary in proportion to its concentration [102].

Acute exposure to stressful stimuli activates the HPA axis as well as sympathetic nervous system, leading to physiological responses, such as elevated blood pressure, an increased ACTH, and corticosterone in the plasma [103,104,105]. However, chronic PS exposure leads to HPA axis alterations such as decreased rise of morning cortisol and increased baseline secretion of cortisol [106]. Moreover, continuous PS is not only linked to the HPA axis, but also to several mediators, such as catecholamines, prolactin, neuropeptides, and nerve growth factors [51,107]. Substance P (SP), which is released from sensitive nervous endings, increases sensory perception and pruritus in the skin [38]. However, it also inhibits the HPA axis and therefore modifies the PS response. In the presence of SP, which triggers a shift towards a chronic PS response, the HPA axis response slows down. Under the chronic exposure to PS, there are several pathological changes in the skin, such as a delay in wound healing [77] and an impairment in skin barrier recovery [108,109]. Chronic PS has been shown to result in a disruption of the skin-barrier, as demonstrated by an increase in TEWL in affected mice [108]. Recent findings have suggested that PS decreases epidermal proliferation and differentiation, disrupts the permeability barrier homeostasis, and decreases the skin integrity of the SC [109]. For example, insomniac PS has been shown to change both barrier homeostasis and SC integrity. Insomniac PS inhibition of epidermal lipid synthesis is correlated to decreased lamellar body formation and decreased corneodesmosomes, thereby negatively affecting barrier homeostasis and altering SC integrity [109]. Aioi et al. demonstrated that overcrowding PS resulted in dry skin and barrier impairment. The dry skin and barrier disruption have been associated with decrease in compounds important to skin barrier function, e.g., ceramide and pyrrolidone carboxylic acid [108]. Moreover, Choi et al. reported that elevated GC concentration could result in permeability barrier changes and delayed barrier recovery after tape stripping, which is linked to decreased epidermal lipid synthesis, as observed during PS [109]. Evidence also suggests that administration of RU-486 (a GC receptor antagonist) or antalarmin (a CRH antagonist) in psychologically stressed mice improves barrier recovery [110]. These results emphasize the importance of GCs induced during PS and their effect on SC homeostasis [109,110], suggesting that GCs play a major role in barrier function under stressed conditions.

Systemic and topical GC applications cause adverse effects on skin structure and function comparable to those observed with PS [109,110]. PS increases the production of endogenous GCs, which are released from the adrenal gland in response to stressful stimuli as part of the activation of HPA axis. Acute PS activates metabolic responses that help the host immediately in the short-term, though the current medical paradigm holds that PS actually worsens systemic and cutaneous inflammatory disorders. Additionally, superimposed exogenous (motion-restricted) stress resulted in a reduction in inflammation and improved skin barrier function in a murine AD model, even going as far as normalizing serum immunoglobulin E (IgE) levels [111]. These benefits are the result of increased endogenous GC concentration, which have been explained because the actions of the HPA axis are normally tightly regulated to ensure that the body can respond quickly to stressful events and return to a normal state just as rapidly. Thus, PS, in the acute phase, may be a beneficial protective mechanism by providing anti-inflammatory effects through the production of endogenous GCs. This is a different view to the current paradigm of PS aggravating cutaneous inflammatory dermatoses [111]. When PS is maintained and sustained over time, the adverse consequences have been shown in AD. Persistent PS may stimulate a rise in endogenous GCs which compromises the permeability barrier homeostasis, SC cohesion, wound healing, and epidermal innate immunity in skin. Persistent exposure of elevated GC burden causes not only body homeostasis disturbance (e.g., blunting the HPA axis response), but also adverse sequelae affecting cognitive processes [85,106].

The link between PS and chronic inflammatory dermatoses such as AD is complex. On the one hand, patients with AD have been shown to have reduced production of cortisol and ACTH by the experimental Trier Social Stress Test when compared with non-atopic controls. AD patients had blunted HPA axis reactivity as assessed by cortisol and ACTH measurements [112,113]. On the other hand, AD can elicit PS and release of GCs [1,3]. Hence, chronic GC elevation disturbs neuroendocrine signaling and can induce neuroinflammation, neurotoxicity, and cognitive impairment [106]. One study assessed the effects of AD-induced stress in a mouse AD model and the results have shown that AD-related PS increased astroglial and microglial activation, neuroinflammatory cytokine expression, and markers of neuronal loss [114].

CRH induces arginine vasopressin secretion, which acts synergistically with CRH, especially under chronic stress state [53,61,115,116]. Moreover, CRH has an inhibitory effect on the pineal secretion of melatonin in normal, unaffected persons [117]. Melatonin and its metabolites have been to be important for proper physiological skin function and effective protection of a cutaneous homeostasis from adverse environmental stimuli, and its use can attenuate hyperproliferative/inflammatory conditions [118]. Research has shown that administering melatonin inhibited skin lesions formation, scratching, and elevation of serum IgE levels in the murine AD stressed models [114]. Furthermore, it caused a significant reduction in CRH responsiveness, and a substantial decrease in neuronal damage [114]. Research has been conducted on healthy adults to evaluate the effect of psychological relaxation on perturbed skin barrier. The results showed that the psychological relaxation has been correlated to a beneficial effect on skin barrier recovery regardless of whether before or after injury [119].

Because of itchiness in AD, scratching may lead to a wound, increasing the risk for a secondary bacterial infection. Physicians have been aware that bacteria and other microorganisms are triggers in the etiology of AD [1,3]. PS has also shown to inhibit AMP production which has been shown to result in increased risk for a severe skin infection [120]. Aberg et al. reported that PS increased the severity of skin infection in mice by decreasing the levels of two important AMPs (cathelin-related AMP and beta-defensin 3) in the epidermis, thereby reducing their delivery into lamellar bodies. This may be attributed to increased production of endogenous GCs in response to stress [120]. Moreover, it has been shown that inhibition of CRH and GCs decreased the severity of infection by allowing AMP levels to increase back to baseline levels [120].

## 7. Conclusions

AD is a chronic inflammatory disease of the skin that results from interplay of environmental stressors and PS response. There is a dual link between AD and PS. On one hand, AD produces PS in patients with the disease. Conversely, AD may also be exacerbated by PS. Currently, the activation of both the central and peripheral cutaneous HPA axes is considered the molecular mediator of PS response, resulting in profound effects on the neuroimmune reaction and skin barrier function. The HPA axis dysfunction has been described in AD patients. Since inflammation and skin barrier function are the two key components of the pathogenetic mechanism of AD, research on PS response is of significant interest. Paradoxically, acute PS, acting through the activation of the HPA axis, displays metabolic responses that provide immediate benefit to the host (demonstrated by murine AD models). Thus, exogenous stressors acting in short duration may be of benefit to inflammation. However, extensive exposure may be harmful to both inflammation and skin barrier function. Therefore, the effects of PS in AD may be either beneficial or harmful, which is dependent on the duration of the stressor or the diverse influences on inflammation and skin barrier function.

## Figures and Tables

**Table 1:** Effects of peripheral HPA on skin barrier.

Elements	Psychological Effects	Impacts on Skin Barrier
ACTH	Melanin production	Enhancement
α-MSH	Melanin production	Enhancement
	Strong anti-inflammatory effects	Protection
β-endorphin	Enhances the epidermal turnover rate	Protection
Cortisol	Acute PS: anti-inflammatory effects	Protection
	Chronic PS: Downregulation of AMPs	Compromising
11β-HSD1	Cortisol production	Delay in wound healing

## Abbreviations

ACTH	Adrenocorticotropic hormone
AD	Atopic dermatitis
AMP	Antimicrobial peptide
α-MSH	Alpha melanocyte-stimulating hormone
CRH	Corticotrophin-releasing hormone
CRH-R	Corticotrophin-releasing hormone receptor
EKO	Epidermal knockout
GC	Glucocorticoid
GR	Glucocorticoid receptor
HPA	Hypothalamic–pituitary–adrenal
IL	Interleukin
IgE	Immunoglobulin E
MCR	Melanocortin receptor
POMC	Pro-opiomelanocortin
PS	Psychological stress
SC	Stratum corneum
SP	Substance P
TEWL	Transepidermal water loss
Th2	T helper cell type 2
TSLP	Thymic stromal lymphopoietin
TNF-α	Tumor necrosis factor alpha
11β-HSD	11 beta-hydroxysteroid dehydrogenase
